# A Conserved Planthopper MATH-BTB Protein Regulates Fecundity in *Nilaparvata legens* Stål

**DOI:** 10.3390/ijms27010219

**Published:** 2025-12-24

**Authors:** Yangshuo Dai, Gu Gong, Shiqi Wang, Yujing Guo, Caili Qiu, Yanfang Li, Longyu Yuan, Hanxiang Xiao, Fengliang Jin, Rui Pang, Zhenfei Zhang

**Affiliations:** 1 Guangdong Provincial Key Laboratory of High Technology for Plant Protection, Plant Protection Research Institute, Guangdong Academy of Agricultural Sciences, Guangzhou 510640, China; daiysh@gdppri.com (Y.D.); gonggu1519@163.com (G.G.);; 2 State Key Laboratory of Green Pesticide, College of Plant Protection, South China Agricultural University, Guangzhou 510642, Chinajflbang@scau.edu.cn (F.J.)

**Keywords:** MATH protein family, rice, planthoppers, evolution, NlMATH3, fecundity

## Abstract

The meprin and TRAF-C homology (MATH) family of proteins plays essential roles in diverse biological processes across eukaryotes. Fecundity is a key determinant underlying the rapid outbreaks of agricultural insect pests. Nevertheless, the potential involvement of MATH proteins in the regulation of fecundity in agriculturally important insects, particularly planthoppers, remains largely uncharacterized. This study identified key members of the MATH protein family that are conserved in planthoppers and involved in the regulation of insect fecundity. A total of 121 identified MATH proteins from 31 insect species were classified into five distinct clades based on protein structures, predominantly represented by the MATH-BTB, MATH-USP7, and MATH-Zf-Box subtypes, which are largely conserved across most agricultural insect species. In planthoppers, the MATH-BTB subtype gene cluster *SfMATH1*–*NlMATH3*–*LsMATH3* constitutes a tripartite collinear gene set conserved across all three species. Among the four ovary-specific expressed *MATH* genes, *NlMATH3* exhibited the highest expression level in the ovary. Moreover, silencing *NlMATH3* significantly impaired ovarian development in adult females and reduced both the number of deposited and hatched eggs, which was associated with downregulation of vitellogenin (*Vg*) and its receptor *VgR*, as well as elevating activity in metabolic and immune signaling pathways. In summary, this study provides novel insights into the evolutionary dynamics of the MATH family in agricultural insects, particularly planthoppers, and elucidates the critical regulatory role of the planthopper conserved MATH-BTB protein NlMATH3 in insect fecundity. The conservation of NlMATH3 homologs across planthoppers highlights their potential as targets for RNAi-based pest control strategies.

## 1. Introduction

The MATH (meprin and TRAF-C homology) family comprises proteins defined by the presence of a conserved MATH domain [1,2,3]. The term “MATH domain” was introduced based on its structural similarity to the C-terminal domain of human meprin metalloproteinases and tumor necrosis factor (TNF) receptor-associated factors (TRAF-C) [4,5,6]. In eukaryotes, most MATH proteins contain additional functional domains, such as the BTB (broad-complex, tramtrack, and bric-a-brac) domain, the ubiquitin-specific protease (USP) domain, and the RING/zinc finger domain. Depending on the domain arrangement, MATH family proteins are classified into several subtypes, including MATH-only, MATH-BTB, MATH-USP7, and MATH-TRIM [3,5,7,8]. In mammals and plants, MATH proteins function as key signaling molecules in various physiological processes, including growth and development and immune responses [5,9,10,11,12,13]. They often act as molecular adaptors that link various receptors to downstream signaling components [4,14,15,16,17]. However, the classification, evolutionary relationships, and functional roles of MATH proteins in insects, particularly in agriculturally significant species, remain largely uncharacterized.

The planthopper is one of the most destructive pests of rice, causing substantial yield losses or even complete crop failure [18,19,20]. The brown planthopper (*Nilaparvata lugens* Stål), the small brown planthopper (*Laodelphax striatellus* Fallén), and the white-backed planthopper (*Sogatella furcifera* Horváth) are three major rice planthopper species. Among them, *N. lugens* is the most damaging due to its significantly higher reproductive capacity [21]. A single female *N. lugens* can lay approximately 100–300 eggs per oviposition event, leading to rapid population outbreaks. Therefore, identifying genes essential for *N. lugens* fecundity and targeting them via RNA interference (RNAi) represents a promising strategy for pest control [22,23,24].

The mechanisms underlying fecundity in *N. lugens* have been investigated from multiple perspectives, including gene regulation [21,25], signaling pathways [26,27,28], lipid metabolism [29,30], and epigenetic regulation [31,32,33]. Vitellogenin (Vg) and the vitellogenin receptor (VgR) play central roles in ovarian development and reproduction [34,35,36]. Moreover, Vg-mediated ovarian development is regulated by the juvenile hormone (JH)–target of rapamycin (TOR)–insulin signaling axis, which integrates nutritional sensing, lipid metabolism, and hormonal signaling [21,26,37,38,39,40,41]. Although numerous fecundity regulators have been identified, the potential role of MATH proteins remains unexplored. Given their function as key signaling adaptors and the reported role of TRAF6 (a MATH-domain protein) in regulating reproduction in the housefly (*Musca domestica*) [42], we hypothesize that NlMATH proteins are involved in modulating *N. lugens* fecundity.

Therefore, this study first systematically identified MATH family members across various insect species to elucidate their evolutionary relationships, with a focus on rice planthoppers. Subsequently, temporal and spatial expression patterns, as well as tissue-specific expression profiles, of all four *N. lugens* MATH genes were investigated to elucidate their potential functional roles. Given that *NlMATH* genes constitute an ovary-enriched gene family and *NlMATH3* exhibits the highest expression level in the ovary along with a stage-specific expression pattern, this planthopper-conserved MATH-BTB protein is presumed to be a key regulator of insect fecundity. Finally, the functional role of *NlMATH3* in regulating ovarian development was confirmed through RNAi, and the underlying mechanism was preliminarily explored via transcriptomic analysis.

## 2. Results

### 2.1. Identification of MATH Family Members in 31 Insect Species

The present study identified MATH domain-containing proteins in agriculturally relevant insect species through a comprehensive genome-wide search using HMMER with the Pfam MATH domain (PF00917) as a query. The analysis encompassed the proteomes of 31 insect species, primarily crop pests, spanning five orders: Diptera (5 species), Hemiptera (8 species), Coleoptera (5 species), Hymenoptera (6 species), and Lepidoptera (7 species). Candidate sequences were further validated for the presence of the MATH domain using the SMART and CDD databases. This approach identified a total of 121 sequences classified as putative members of the MATH protein family across the analyzed species (Table S1).

Domain architecture analysis revealed that, in addition to the conserved MATH domain, these proteins contain four associated domains: USP7, UCH, BTB, and Zinc finger-Box (Zf-Box) (Table S1). Specifically, MATH-BTB, MATH-USP7, and MATH-Zf-Box domain architectures were detected in 30, 29, and 25 insect species, respectively (Table S1). The number of MATH-BTB proteins per species ranged from one to ten, positively correlating with the overall number of MATH proteins in each species. In contrast, most species possessed only a single copy of MATH-USP7 and MATH-Zf-Box proteins, with the exception of *Belonocnema treatae*, which harbors two MATH-Zf-Box proteins (Table S1). These results suggest that MATH-BTB, MATH-USP7, and MATH-Zf-Box represent the predominant domain architectures among MATH proteins in agriculturally relevant insects, with MATH-BTB proteins likely driving the expansion of this family in certain lineages.

### 2.2. Phylogenetic Clustering of the 121 Identified MATH Family Members

**Figure 1:**
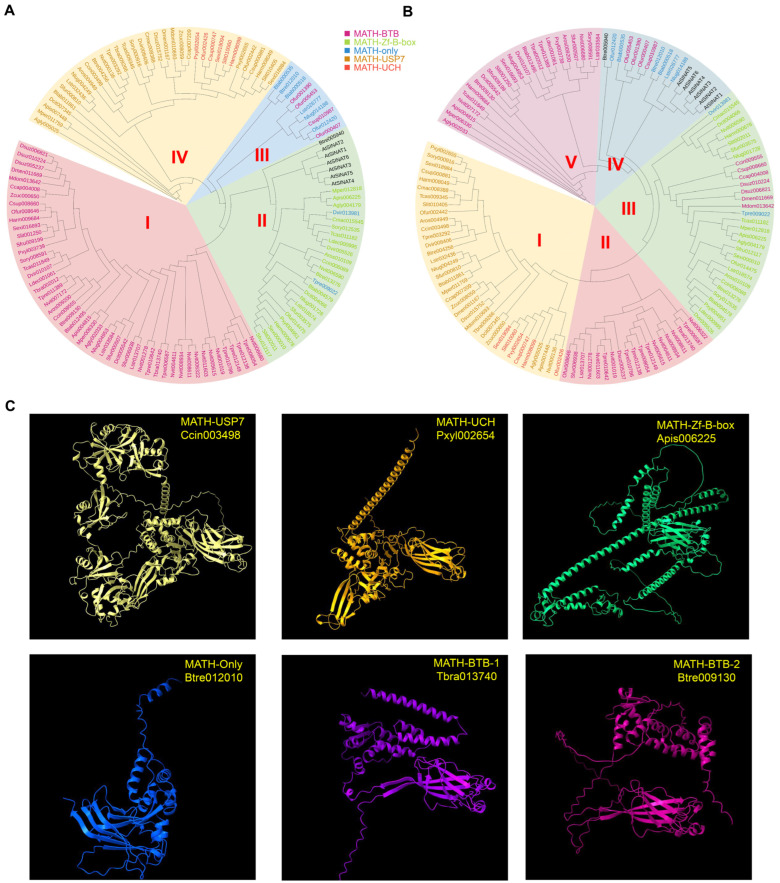
Identification and phylogenetic clustering of MATH family members in 31 insect species. (**A**) Unrooted phylogenetic tree of insect MATH proteins. Phylogenetic relationships were derived using the maximum likelihood method and JTT matrix-based model in MEGA software (v12). I, MATH–BTB; II, MATH–Zf–B–box; III, MATH–Only; IV, MATH–USP7 and –UCH. (**B**) The classification of proteins into different MATH families based on protein structure and labeled with different color modes. I, MATH–USP7 and –UCH; II, MATH–BTB–1; III, MATH–Zf–B–box; IV, MATH–Only; V, MATH–BTB–2. (**C**) Representative predicted structures for each of 5 MATH clades.

To investigate the phylogenetic relationships among the 121 identified MATH family members, a ML phylogenetic tree was constructed based on their amino acid sequences. The analysis grouped the MATH proteins into four distinct clades (Figure 1A), which are largely consistent with their functional domain architectures. All proteins in Group I were MATH-BTB proteins, while all MATH-Zf-Box proteins, together with two MATH-only proteins, clustered in Group II. The MATH-USP7 and MATH-UCH proteins were classified into Group IV, as MATH-USP7 proteins typically contain a MATH-UCH domain at the N-terminus and USP7 domains at the C-terminus. Additionally, the remaining MATH-only proteins were clustered with four MATH-BTB proteins in Group III, likely due to limited representation of this subtype.

Given that three-dimensional (3D) structure-based clustering has proven effective for protein family classification [43], a structure-informed clustering approach was applied to validate these groupings. Full-length protein sequences of the 121 MATH members were subjected to structure prediction using AlphaFold2. Based on these predicted structures, similarity matrices were generated through MSAs (Figure S1), and a structural dendrogram was subsequently constructed using UPGMA (Figure 1B). Similar to the 2D sequence-based phylogenetic tree, this dendrogram classified the MATH-USP7 and MATH-UCH proteins, all MATH-Zf-Box proteins, and the MATH-only proteins into three distinct clusters. However, the MATH-BTB proteins were primarily divided into two separate clades: Group V included MATH-BTB proteins from insect species containing one or two members of this subtype, while Group II comprised those from two parasitic wasp species harboring more than six members. Moreover, the 3D protein structures across these MATH subtypes exhibit substantial structural divergence, which is also evident between the two MATH-BTB subgroups (Figure 1C). These findings collectively indicate that the three predominant MATH protein subtypes—MATH-BTB, MATH-USP7, and MATH-Zf-Box—are largely conserved across most agricultural insect species, although the MATH-BTB subtype may have undergone expansion in certain evolutionary lineages.

### 2.3. Characterization and Evolutionary Analysis of Planthopper MATH Members

**Figure 2:**
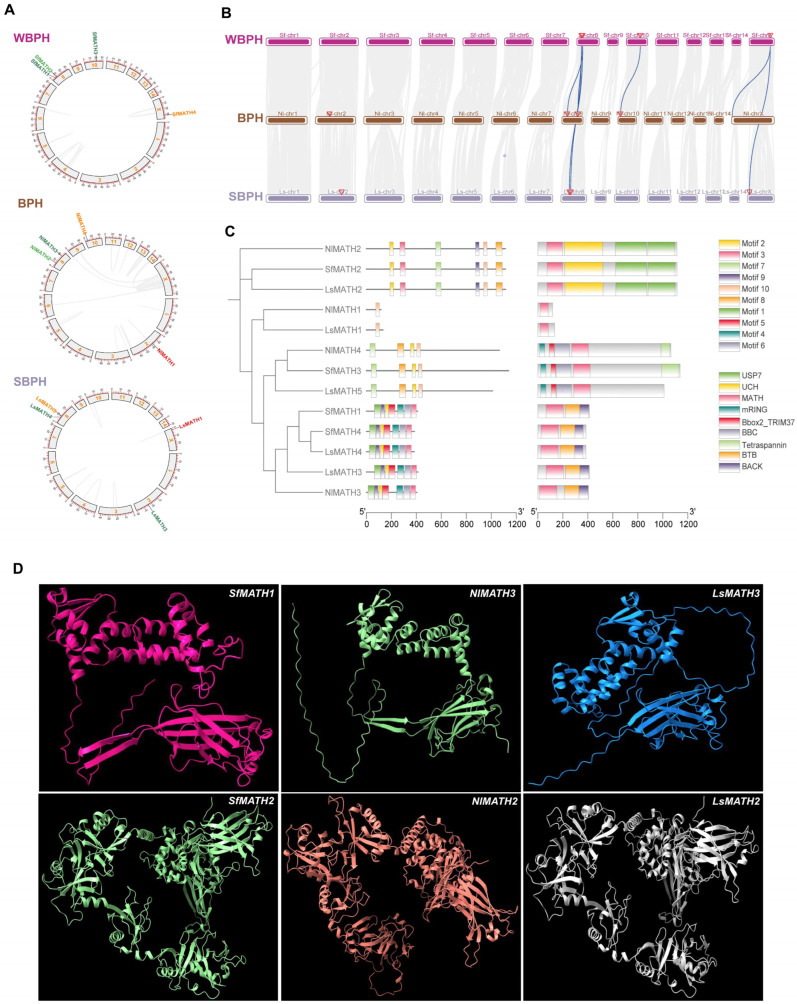
Characterization and evolutionary analysis of planthopper MATH members. (**A**) Chromosome mapping, gene duplication, and synteny analyses of MATH genes in rice planthoppers. (**B**,**C**) Phylogeny (**B**) and domain organization (**C**) of MATH proteins of rice planthopper (*Sogatella furcifera*, *Nilaparvata lugens*, and *Laodelphax striatellus*). “Δ”indicates the position of *MATH* genes in corresponding specie genomes). (**D**) 3D structure of two collinear gene clusters in planthoppers, *SfMATH1*–*NlMATH3*–*LsMATH3* and *SfMATH2*–*NlMATH2*–*LsMATH2*.

Among the 121 identified MATH proteins across insect species, 13 sequences were derived from three rice planthopper species: four from *N. lugens* (NlMATH), four from *S. furcifera* (SfMATH), and five from *L. striatellus* (LsMATH). Based on their chromosomal positions, the genes encoding these 13 MATH proteins were designated as *NlMATH1–4*, *SfMATH1*–*4*, and *LsMATH1–5* for *N. lugens*, *S. furcifera*, and *L. striatellus*, respectively (Figure 2A, Table S2). Detailed characterization revealed that the planthopper MATH proteins ranged in length from 118 amino acids (aa) (NlMATH1) to 1139 aa (SfMATH3), with molecular weights (Mw) ranging from 13.59 kDa to 124.5 kDa, and theoretical isoelectric points (pI) varying between 5.41 (SfMATH2) and 10.00 (NlMATH1) (Table S2). Furthermore, among these 13 MATH proteins, five were classified as MATH-BTB proteins (NlMATH3, SfMATH1, SfMATH4, LsMATH3, and LsMATH4), three as MATH-USP7 proteins (NlMATH2, SfMATH2, and LsMATH2), three as MATH-Zf-Box proteins (NlMATH4, SfMATH3, and LsMATH5), and two as MATH-only proteins (NlMATH1 and LsMATH1) (Table S2). In contrast to the expansion of the MATH family—particularly the MATH-BTB subfamily—observed in other insect species such as *Ostrinia furnacalis* and *Nasonia vitripennis*, rice planthoppers exhibit a contracted MATH family.

Further, intraspecific collinearity analysis was performed for the three planthopper species and revealed no evidence of tandem or segmental duplication events among these *MATH* genes (Figure 2B). In contrast, interspecific collinearity analysis across the three species identified nine orthologous MATH gene pairs, organized into three conserved syntenic blocks: three between *S. furcifera* and *N. lugens* (*SfMATH1*–*NlMATH3*, *SfMATH2*–*NlMATH2*, and *SfMATH3*–*NlMATH4*), three between *S. furcifera* and *L. striatellus* (*SfMATH1*–*LsMATH3*, *SfMATH2*–*LsMATH2*, and *SfMATH4*–*LsMATH5*), and three between *N. lugens* and *L. striatellus* (*NlMATH1*–*LsMATH1*, *NlMATH2*–*LsMATH2*, and *NlMATH3*–*LsMATH3*) (Figure 2B). Additionally, two tripartite collinear gene sets were detected—*SfMATH1*–*NlMATH3*–*LsMATH3* and *SfMATH2*–*NlMATH2*–*LsMATH2*—each representing a single MATH gene with conserved synteny across all three species (Figure 2B). Functional domain analysis revealed that the proteins in the *SfMATH1*–*NlMATH3*–*LsMATH3* cluster belong to the MATH-BTB subtype, whereas those in the SfMATH2–NlMATH2–LsMATH2 cluster are of the MATH-USP7 subtype (Figure 2C). Moreover, predicted three-dimensional (3D) structures showed high structural similarity among all members of each orthologous triplet (Figure 2D). These findings suggest that the MATH genes in each collinear gene cluster, *SfMATH1*–*NlMATH3*–*LsMATH3* and *SfMATH2*–*NlMATH2*–*LsMATH2*, originated from a common ancestor shared by the three species.

### 2.4. The Spatiotemporal Expression Patterns of the NlMATH Genes

**Figure 3:**
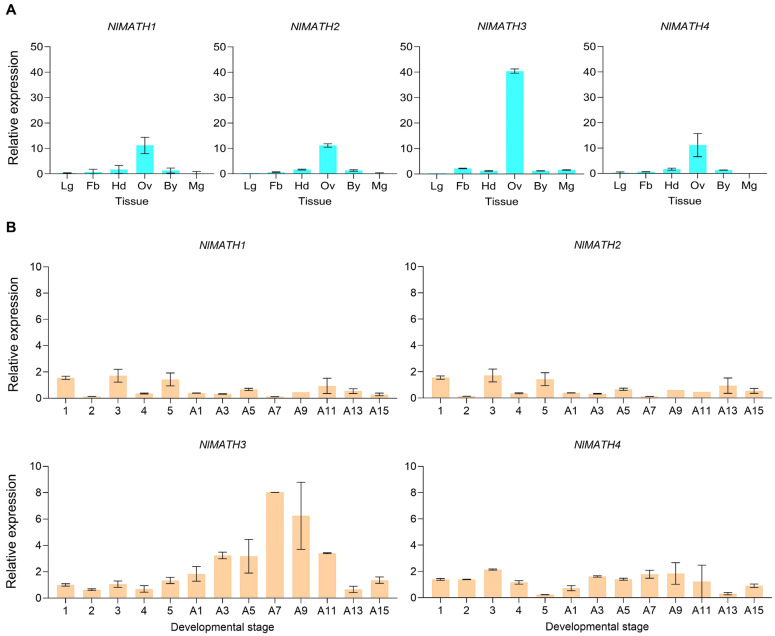
The spatiotemporal expression patterns of four NlMATH genes in *N. lugens*. MATH genes (*NlMATH1*, *NlMATH2*, *NlMATH3*, *NlMATH4*) in whole bodies at various developmental stages and in different tissues. (**A**) Expression levels of four NlMATH genes (*NlMATH1*, *NlMATH2*, *NlMATH3*, *NlMATH4*) in different tissues (Lg, leg; Fb, fat body; Hd, head; Ov, ovary; By, body; Mg, midgut) of *N. lugens* female adults. (**B**) Expression profiles of four NlMATH genesRNA at various developmental stages, including nymphs at 1–5 days and female brachypterous brown planthoppers at 1, 3, 5, 7, 9, 11, 13, and 15 days. The panels show the mean transcript levels ± SD (*n* = 3 biological replicates).

To elucidate the potential functional roles of MATH members in rice planthoppers, we selected *N. lugens* as a representative species, given its status as the most destructive pest among the three planthopper species. The spatiotemporal expression profiles of four *NlMATH* genes were analyzed using qRT-PCR across six distinct tissues (head, midgut, ovary, fat body, leg, and body) and 13 developmental stages [five nymphal stages (N1–N5) and eight adult stages (A1–A15)]. Expression of all four *NlMATH* genes was detected in all examined tissues, with higher transcript levels observed in the ovary compared to the other five tissues, particularly for *NlMATH3* (Figure 3A). This finding suggests that *NlMATH* genes constitute an ovary-enriched gene family. Notably, *NlMATH3* transcript levels remained stable during the nymphal stages (N1–N5), gradually increased during the early adult stage (A1–A9), and subsequently declined at later adult stages (A11–A15) (Figure 3B). In contrast, such a stage-specific expression pattern was not observed for the other three *NlMATH* genes (Figure 3B). These results indicate a potential role for *NlMATH3* in regulating fecundity in *N. lugens*.

### 2.5. Fecundity Analysis of NlMATH3 Using RNAi

**Figure 4:**
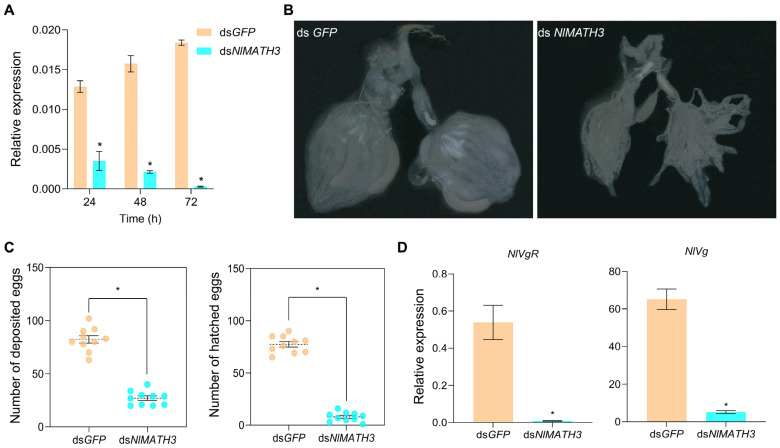
Fecundity analysis of NlMATH3 using RNAi. (**A**) The expression level of *NlMATH3* in *N. lugens* at 24 h–72 h after injected with specific dsRNA (ds*GFP* and ds*NlMATH3*). Data are means ± SD (*n* = 3 biological replicates). (**B**) Anatomical map of ovary in *N. lugens* after injected ds*GFP* or ds*NlMATH3*. (**C**) the oviposition behaviors of *N. lugens* were determined every 24 h. Data are means ± SD (*n* = 10 biological replicates). (**D**) The expression level of two fecundity-related genes (*NlVgR*, *NlVg*) in *N. lugens* female adults at 72 h after injected with ds*NlMATH3*. Data are means ± SD (*n* = 3 biological replicates). Asterisks (*) indicate significant difference between two groups conducted by *t*-test, *p* < 0.05.

To validate the role of *NlMATH3* in regulating ovarian development in *N. lugens*, RNA interference (RNAi) by microinjecting dsRNA targeting *NlMATH3* (ds*NlMATH3*) was performed into adult female *N. lugens* to silence the gene. qRT-PCR analysis revealed that *NlMATH3* transcript levels were significantly reduced at 24 h (65%), 48 h (79%), and 72 h (98%) post-injection compared to the control group (ds*GFP*) (Figure 4A), confirming efficient knockdown of *NlMATH3*. Observation of dissected ovaries revealed that silencing of *NlMATH3* largely impaired ovarian development in adult female *N. lugens* (Figure 4B). Notably, the number of eggs laid and the hatching rate decreased by 67.07% and 89.66%, respectively, in the ds*NlMATH3* group compared to the ds*GFP* group from the third day until the fifteenth day after injection (Figure 4C). In addition, the expression levels of *NlVg* and *NlVgR*—two genes involved in regulating fecundity in *N. lugens*—were substantially downregulated (by 92.1% and 99.5%, respectively) at 72 h post-RNAi (ds*NlMATH3*) relative to the control (ds*GFP*) (Figure 4D). These findings confirm that *NlMATH3* plays an essential role in regulating *N. lugens* fecundity.

### 2.6. Transcriptomic Analysis of N. lugens Females After NlMATH3 Silencing

**Figure 5:**
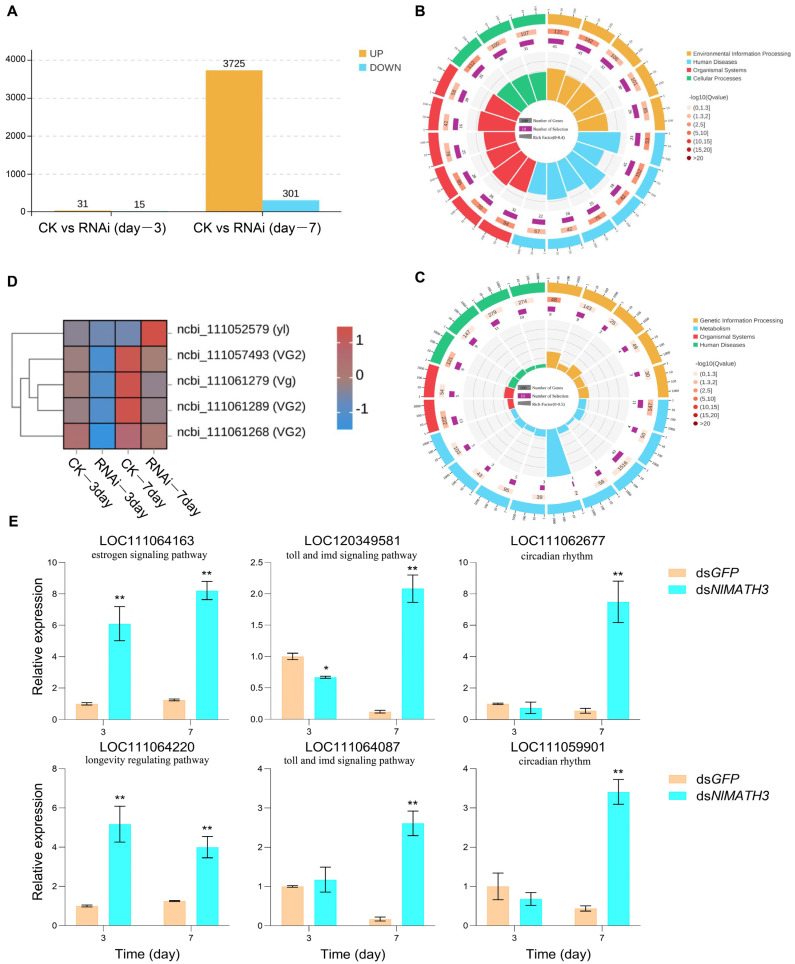
Transcriptomic analysis of *N. lugens* females after *NlMATH3* silencing. (**A**) The number of differential genes at 3 and 7 days after RNA interference. (**B**,**C**) Kyoto Encyclopedia of Genes and Genomes (KEGG) pathway enrichment analysis of differentially expressed genes (DEGs) that were upregulated (**B**) and downregulated (**C**) by RNA interference for 7 days. (**D**) Heatmap of expression levels of fecundity-related genes in *N. lugens* at 3 and 7 days after RNA interference. (**E**) The expression levels of six DEGs associated with immune pathways and circadian rhythm regulation in *N. lugens* at 3 and 7 days after RNA interference validated by qRT-PCR. Data are means ± SD (*n* = 3 biological replicates). Asterisks indicate significant difference between two groups conducted by *t*-test, * *p* < 0.05, ** *p* < 0.01.

To further elucidate the effects of *NlMATH3* on *N. lugens* fecundity, transcriptomes of females treated with ds*GFP* (CK) and ds*NlMATH3* (RNAi) were sequenced at two time points—day 3 and day 7 after microinjection. Interestingly, compared with the day-3 group (46 DEGs), the day-7 group exhibited a significantly higher number of differentially expressed genes (4026 DEGs), comprising 3725 upregulated and 301 downregulated genes (Figure 5A). These findings indicate a progressive and accumulating regulatory role for *NlMATH3* during *N. lugens* development. The upregulated DEGs in the day-3 group were primarily enriched in pathways related to longevity regulation, estrogen signaling, and MAPK signaling (Figure S2A). In contrast, downregulated DEGs in the day-3 group were associated with vitamin digestion and absorption, ovarian steroidogenesis, cholesterol metabolism, and fat digestion and absorption (Figure S2B). The upregulated DEGs in the day-7 group were predominantly enriched in pathways including MAPK signaling, cAMP signaling, dopaminergic synapse, and insulin secretion (Figure 5B). In contrast, downregulated DEGs in the day-7 group were linked to thermogenesis, oxidative phosphorylation, and fatty acid metabolism (Figure 5C).

In addition, fecundity-related genes in *N. lugens*—including *Vg* (ncbi_111061279), *Vg-like* (ncbi_111057493, ncbi_111061289, ncbi_111061268), and *YL* (ncbi_111052579)—were reanalyzed using transcriptomic datasets. The heatmap showed that expression levels of these genes in the CK group were higher on day 7 than on day 3 (Figure 5D). Moreover, the expression levels of *Vg* and *Vg-like* genes in the RNAi group were markedly lower than those in the CK group at both day 3 and day 7; however, the *YL* gene exhibited an opposite trend (Figure 5D). These results confirm that RNAi-mediated silencing of *NlMATH3* effectively modulates fecundity-associated genes in *N. lugens.*

Finally, the expression levels of six DEGs associated with immune pathways and circadian rhythm regulation were analyzed by qRT-PCR to validate the transcriptomic data generated in this study. The qRT-PCR results revealed significantly higher expression levels of these genes in the ds*NlMATH3* group compared to the ds*GFP* group on day 7, consistent with the transcriptome data (Figure 5E). All the above data are represented as the mean ± SD from 10 biological replicates, asterisks (*) indicate significant difference (* *p* < 0.05, ** *p* < 0.01 *t*-test).

## 3. Discussion

MATH family proteins play key regulatory roles in various biological processes in mammals and plants; however, information regarding the evolutionary relationships and potential functional roles of this protein family in agriculturally relevant insects remains largely unknown. This study provides a comprehensive evolutionary and functional analysis of the MATH protein family in agricultural insects, revealing the conservation of key subtypes and identifying the MATH-BTB protein NlMATH3 as a critical regulator of fecundity in *N. lugens*.

In plants, dozens of MATH members have been identified in individual species, including Arabidopsis, rice, and Solanaceae family [5,7]. In contrast, this study found that MATH proteins across 31 insect species from five orders comprise 2 to 5 members per species, with the exception of three species. Moreover, unlike the four subtypes found in plants, single-MATH, MATH-USP7, MATH-BTB, and multiple-MATH [7,41], this study categorizes insect MATH proteins into five subtypes: MATH-BTB, MATH-USP7, MATH-Zf-Box, MATH-only, and MATH-UCH. Among these, MATH-BTB, MATH-USP7, and MATH-Zf-Box represent the predominant subtypes, typically comprising one to two members in most insect species. In addition, multiple MATH-BTB members were detected in only three insect species, consistent with the expansion of MATH members in these lineages—a phenomenon also observed in plant species where MATH-BTB gene family expansion is a major evolutionary mechanism [7]. Collectively, these findings suggest that the numbers of MATH-BTB, MATH-USP7, and MATH-Zf-Box subtypes are likely conserved across most agricultural pest insect species.

The 3D structure and folding of proteins play crucial roles in determining protein function [43]. In this study, predicted 3D structures based on AI methods [44,45] were compared and clustered to classify the proteins [46]. By integrating phylogenetic analysis with structure-based clustering of protein 3D conformations, the 121 identified MATH proteins were classified into five clades, as opposed to four clades in the 2D sequence-based phylogenetic tree. Unlike the single clade of MATH-BTB proteins observed in the 2D phylogenetic tree, the structural dendrogram resolved these proteins into two distinct clades. This separation clearly distinguishes conserved MATH-BTB members from most agricultural insect species from the expanded copies found in two parasitic wasp species. This comparison supports the conservation of the MATH-BTB, MATH-USP7, and MATH-Zf-Box subtypes across most agricultural pest insect species. Furthermore, the identification of conserved collinear gene clusters—the MATH-BTB cluster *SfMATH1*–*NlMATH3*–*LsMATH3* and the MATH-USP7 cluster *SfMATH2*–*NlMATH2*–*LsMATH2*—across the three planthopper species provides additional evidence for the evolutionary conservation of the MATH protein family in agricultural insects. Thus, this study provides further support for the accuracy of protein 3D structure in protein classification.

Interestingly, spatiotemporal expression profiling revealed an enrichment of *NlMATH3* in the ovary of *N. lugens*, with its transcript levels peaking during early adulthood, coinciding with ovarian maturation. Furthermore, RNAi-mediated silencing of *NlMATH3* significantly impaired ovarian development and reduced the number of deposited and hatched eggs in *N. lugens.* These RNAi-treated females exhibited low expression levels of *NlVg* and *NlVgR* genes, which are key regulators of vitellogenesis and ovarian development [21,34,35,36]. In *N. lugens*, the JH-TOR-insulin signaling axis is a key node that integrates nutritional sensing, lipid metabolism, and hormonal signaling to synergistically regulate Vg synthesis and fecundity [21,25]. JH directly regulates Vg protein synthesis by activating genes associated with the biosynthetic pathway [26,37,38,39,47], and also indirectly regulates Vg synthesis through the activation of insulin-like peptides (ILPs) [41]. The TOR pathway promotes JH synthesis by sensing amino acid availability and indirectly modulates Vg expression by regulating GATA transcription factors and ILPs [27,28,34]. In this study, transcriptomic analysis revealed that *NlMATH3* silencing not only reduced the transcript levels of genes related to vitellogenesis and lipid metabolism, but also led to the upregulation of genes involved in the MAPK signaling, cAMP signaling, and insulin signaling pathways. Given that MATH proteins typically function as key molecular adaptors linking different signaling pathways to regulate physiological processes [5,9,11,13], these findings suggest that NlMATH3 may function as an important regulator in the fecundity of *N. lugens.* In this stuy, only the pathway affected following NlMATH3 interference were analyzed via transcriptome sequencing, whereas the interacting proteins of NlMATH3 were not further identified, nor were their functions elucidated. Therefore, the precise molecular mechanisms by which NlMATH3 regulates *Vg*/*VgR* expression and thereby influences insect fecundity—whether this occurs via the JH-TOR-insulin signaling axis—remain to be further elucidated.

The functional significance of *NlMATH3* in planthopper fecundity opens novel avenues for developing targeted pest management strategies based on the RNAi approach. In the past few decades, RNAi has been widely used to elucidate the functions of genes involved in pest development and to screen potential targets for genetic manipulation [24]. In *N. lugens*, injection with *dsNlVgR* and *dsTOR* inhibited spawning and ovarian development [27,34], while silencing of *NLInR1* and *NLInR2* reduced *Vg* and *VgR* expression and impaired ovarian development [48]. In this study, *dsMATH3*-treated *N. lugens* exhibited sustained high RNAi efficiency even after 7 days, resulting in significantly reduced mRNA levels of *Vg* and *VgR*, along with impaired ovarian development and a reduction in egg production. Combined with the presence of the tripartite collinear gene set *SfMATH1*–*NlMATH3*–*LsMATH3* across three planthopper species, *NlMATH3* and its homologs could serve as a promising RNAi target for suppressing planthopper populations in the future. Furthermore, the structural conservation of the MATH-BTB subtype among agricultural insect species highlights that insights gained from planthoppers may be applicable to other agricultural pests.

## 4. Materials and Methods

### 4.1. Insect Strains

The *N. lugens* population used in this study is an inbred line originally collected from field populations and has been maintained on the susceptible rice variety Taichung Native (TN1) rice seedlings in a growth chamber (27 ± 2 °C, 77% ± 5% relative humidity, and a 12-h:12-h light/dark photoperiod) for over five years. The fecundity of this population has remained consistently within normal ranges over an extended period, despite the absence of routine microbiota screening.

### 4.2. Identification and Characterization of Insect MATH Members

The HMM profile of the MATH domain (Pfam:PF00917) was used as a query to identify candidate proteins in 31 insect species using HMMER 3.0 with an E-value cutoff of 10^−5^ [49]. Genome sequences were obtained from InsectBase 2.0 (http://v2.insect-genome.com) (accessed on 7 September 2025). Each non-redundant candidate was validated for the presence of a complete MATH domain. Additional conserved functional domains within the protein sequences were identified using the SMART and CDD databases. Molecular weight (Mw) and theoretical isoelectric point (pI) were predicted using ExPASy Prosite (https://prosite.expasy.org) (accessed on 10 September 2025). Conserved motifs of planthopper MATH proteins were identified using the MEME Suite (v5.5.2) [50].

### 4.3. Prediction of Protein 3D Structures and Phylogenetic Tree Construction

Protein 3D structure prediction and phylogenetic analysis were performed as previously described [43]. High-confidence 3D structures of MATH proteins were predicted using AlphaFold2. Only models with an average per-residue confidence score (pLDDT) ≥ 70 were retained after filtering to ensure structural reliability. Structural representations were generated using PyMOL (v2.2.5). Pairwise structural alignments were conducted using the TM-score method, which accounts for the relative distances between corresponding residues. The Min-Max normalization method was applied to scale the raw similarity scores into a uniform range of [0, 1]. The calculation was carried out using the following formula:

$$x^{\prime }=\frac{x-min\left(X\right)}{max\left(X\right)-min\left(X\right)}$$

In this formula, x represents the raw similarity score, min(X) and max(X) denote the minimum and maximum scores in the entire matrix, respectively, and x′ is the resulting normalized value. The normalized matrix was subsequently clustered via the UPGMA algorithm, and the resulting dendrogram was visualized through iTOL (Interactive Tree of Life, https://itol.embl.de) (accessed on 21 September 2025).

For 2D sequence-based phylogenetic analysis, full-length MATH protein sequences were aligned using MUSCLE with the BLOSUM30 protein weight matrix and default gap extension penalties. Unrooted phylogenetic trees were constructed using MEGA software employing the maximum likelihood (ML) method under the Jones–Taylor–Thornton (JTT) matrix-based evolutionary model, with pairwise deletion of gaps and 1000 bootstrap replicates. The resulting trees were subsequently refined and visualized using iTOL.

### 4.4. Duplication Events and Synteny Analysis

The complete genome sequences and annotation files of *N. lugens*, *S. furcifera* and *L. striatellus* were obtained from InsectBase 2.0 database (http://v2.insect-genome.com) (accessed on 7 September 2025) [51] and used for synteny analysis with MCScanX [52] to identify intragenomic syntenic blocks—indicative of whole-genome duplications—and intergenomic syntenic blocks between related species among *MATH* genes in planthoppers. The classification of duplication events was conducted using the duplicate_gene_classifier program implemented in the MCScanX package. The criteria were defined as follows: tandem duplication refers to a series of consecutive *MATH* genes located on the same chromosome and positioned adjacent to one another; segmental duplication was identified based on intragenomic syntenic blocks detected by MCScanX, and further validated by assessing collinearity conservation within a total of ten flanking genes (five upstream and five downstream) surrounding the target MATH gene. Duplication events and syntenic relationships were visualized using TBtools (v2.119) [53].

### 4.5. RNA Extraction and Real-Time Quantitative PCR (RT-qPCR) Analysis

Total RNA was isolated from different tissues of *N. lugens* female adults (head, body, midgut, ovary, leg, and fat body) and from different developmental stages using the TRIzol reagent (Takara, Dalian, China). First-strand cDNA was synthesized using a cDNA Synthesis SuperMix kit with gDNA Eraser (TransGen Biotech, Beijing, China) according to the manufacturer’s instructions. RT-qPCR was performed on a CFX96 Real-Time PCR system (Bio-Rad, Hercules, CA, USA) with SYBR qPCR Master Mix (Vazyme, Nanjing, China) following the manufacturer’s protocol. The relative transcript levels of target genes were quantified using the 2^−ΔΔCt^ method, with *β*-actin gene serving as an internal reference gene. Primers used in this study are listed in the Table S3.

### 4.6. RNAi and Bioassay of Fecundity in N. lugens Following dsRNA Injection

An approximately 200-bp nucleotide sequence specific to the target gene was amplified. The PCR product was cloned into the pEASY^®^ Blunt Zero vector (TransGen Biotech, Guangzhou, China), and double-stranded RNA (dsRNA) was synthesized from PCR-generated DNA templates using the MEGAscript T7 Transcription Kit (Thermo Fisher, Waltham, MA, USA). To obtain female adult population for RNAi treatment, the fifth-instar *N. lugens* nymphs were transferred to individual rice plants. Every day, newly emerged adults were collected daily as the one-day-old brachypterous individuals, and sexed based on the presence of ovipositors on the abdomen. These female adults were then selected for microinjection with approximately 200 ng of *dsNlMATH3* or *dsNlGFP* (control) using a Nanoject II Auto-Nanoliter Injector (Drummond Scientific, Broomall, PA, USA) as previously described [54]. Injected insects were reared on TN1 rice plants in a climate-controlled chamber. Three independent replicates were performed for each treatment, with approximately 100 *N. lugens* individuals injected per replicate.

Following RNAi treatment, dsRNA-injected females were paired with two treated males and transferred onto TN1 rice plants to assess reproductive performance. At 72 h post-injection, adult females were randomly sampled for ovarian dissection to evaluate ovarian development under a digital microscope (VHX-7000, KEYENCE, Osaka, Japan). The number of newly hatched nymphs per female was recorded daily, and all nymphs were removed each day until no new hatchings occurred over three consecutive days. Subsequently, rice seedlings were carefully dissected to count unhatched eggs. The total number of eggs deposited per female was calculated as the sum of the hatched nymphs and the unhatched eggs.

### 4.7. Transcriptomic Analysis

Approximately 20 *N. lugens* adults per sample were collected from the treatment group (ds*NlMATH3*) and the control group (*dsGFP*) on the third and seventh days after dsRNA injection, and total RNA was extracted using the TRIzol reagent. Subsequently, 1 μg of high-quality RNA was used to construct a cDNA library with the TruSeq RNA Sample Preparation Kit v2 (Illumina, San Diego, CA, USA) according to the manufacturer’s instructions. The cDNA libraries were then sequenced on an Illumina platform at Gene Denovo Bioinformatics Technology Co., Ltd. (Guangzhou, China). All experiments included three biological replicates.

The transcriptome data were mapped to the *N. lugens* reference genome (https://www.ncbi.nlm.nih.gov/datasets/genome/GCF_014356525.2/) (accessed on 20 September 2025) using HISAT2. Subsequently, gene expression levels were calculated using fragments per kilobase of exon model per million mapped reads (FPKM). Differential gene expression between the ds*GFP* and ds*NlMATH3* groups was analyzed using the DESeq2 R package (v4.4.0). The *p*-values were adjusted using the Benjamini–Hochberg method. A corrected *p*-value threshold of 0.05 and an absolute |log2(fold change)| ≥ 1 were applied to identify differentially expressed genes (DEGs).

### 4.8. Statistical Analysis

The differences in mRNA levels of target genes, as well as the numbers of deposited and hatched eggs, between treatment groups were analyzed using *t*-test in SPSS software (v17.0; SPSS Inc., Chicago, IL, USA). All results are presented as mean values ± SD from independent replicates, and differences were considered statistically significant at * *p* < 0.05 and ** *p* < 0.01.

## 5. Conclusions

In summary, this study elucidates the evolutionary conservation of the MATH protein family in agricultural insects, particularly in planthoppers, and identifies the MATH-BTB protein NlMATH3 as a crucial regulator of fecundity in *N. lugens*. NlMATH3 is essential for ovarian development and egg production, likely through regulating vitellogenin expression and modulating key metabolic and signaling pathways. The conservation of NlMATH3 homologs across planthoppers highlights their potential as targets for RNAi-based pest control strategies.

## Data Availability

The original contributions presented in this study are included in the article. Further inquiries can be directed to the corresponding author.

## Supplementary Materials

The following supporting information can be downloaded at: https://www.mdpi.com/article/10.3390/ijms27010219/s1.
