# Supplementary material for: A Conserved Planthopper MATH-BTB Protein Regulates Fecundity in Nilaparvata legens Stål
Source: Int J Mol Sci. 2025 Dec 24;27(1):219. doi: 10.3390/ijms27010219 (PMC12785250; doi:10.3390/ijms27010219)
Supplement: Supplementary file 1 [file ijms-27-00219-s001.zip › ijms-4042029-supplementary.pdf]

Table S1. Comprehensive MATH members identified in 31 insect species

Insect species	Order	Genome Size (Mb)	Total	MATH-BTB	MATH-USP7	MATH-Zf-Box	MATH-only	MATH-UCH
Ceratitis capitata	Diptera	421	2	1	1	0	0	0
Drosophila melanogaster	Diptera	138	2	1	1	0	0	0
Drosophila suzukii	Diptera	258	4	3	1	0	0	0
Musca domestica	Diptera	726	2	1	1	0	0	0
Zeugodacus cucurbitae	Diptera	362	2	1	1	0	0	0
Acyrthosiphon_pisum	Hemiptera	525	3	1	1	1	0	0
Aphis glycines	Hemiptera	292	3	1	1	1	0	0
Bemisia tabaci	Hemiptera	594	5	1	1	1	2	0
Diaphorina citri	Hemiptera	491	3	1	1	1	0	0
Myzus persicae	Hemiptera	335	3	1	1	1	0	0
Nilaparvata lugens	Hemiptera	1062	4	1	1	1	1	0
Sogatella furcifera	Hemiptera	524	4	2	1	1	0	0
Laodelphax striatellus	Hemiptera	626	5	2	1	1	1	0
Callosobruchus maculatus	Coleoptera	974	2	0	1	1	0	0
Diabrotica virgifera	Coleoptera	2404	4	1	1	1	1	0
Leptinotarsa decemlineata	Coleoptera	623	2	1	0	1	0	0
Sitophilus oryzae	Coleoptera	744	3	1	1	1	0	0
Tribolium castaneum	Coleoptera	160	3	1	1	1	0	0
Athalia rosae	Hymenoptera	150	3	1	1	1	0	0
Belonocnema treatae	Hymenoptera	1522	5	1	1	2	1	0
Cephus cinctus	Hymenoptera	156	3	1	1	1	0	0
Nasonia vitripennis	Hymenoptera	287	12	10	1	1	0	0
Trichogramma brassicae	Hymenoptera	227	3	2	1	0	0	0
Trichogramma pretiosum	Hymenoptera	181	9	7	1	1	0	0
Chilo suppressalis	Lepidoptera	789	4	2	1	0	0	1
Helicoverpa armigera	Lepidoptera	325	4	1	1	1	0	1
Ostrinia furnacalis	Lepidoptera	423	8	4	1	1	1	1
Plutella xylostella	Lepidoptera	331	4	1	1	1	0	1
Spodoptera exigua	Lepidoptera	431	4	1	1	1	0	1
Spodoptera frugiperda	Lepidoptera	463	2	1	0	1	0	0
Spodoptera litura	Lepidoptera	424	4	1	1	1	0	1
**Total**	31	-	121	54	29	25	7	6

Table S2. Gene information and protein characteristics of MATH members in planthoppers

Gene name	Protein ID	Gene position located on chromosome	Amino acids (aa)	Mw (kDa)	Isoelectric point (pI)	MATH type
Nilaparvata lugens						
NlMATH1	Nlug014188.1	Chr2 35138755-35141354	118	13597.72	10.0	MATH-only
NlMATH2	Nlug004249.1	Chr8 13673749-13712380	1113	128269.70	5.52	MATH-USP7
NlMATH3	Nlug004953.1	Chr8 39479628-39498413	406	45773.47	5.71	MATH-BTB
NlMATH4	Nlug001728.1	Chr10 6398260-6428280	1065	116568.77	5.67	MATH-Zf-Box
Sogatella furcifera						
SfMATH1	Sfur000507.1	Chr8 6885650-6956825	402	46374.52	8.59	MATH-BTB
SfMATH2	Sfur000810.2	Chr8 8631693-8667597	1114	128808.93	5.41	MATH-USP7
SfMATH3	Sfur003575.1	Chr10 20694774-20723194	1139	124514.54	5.48	MATH-Zf-Box
SfMATH4	Sfur006938.1	ChrX 31663943-31669823	385	43481.67	5.71	MATH-BTB
Laodelphax striatellus						
LsMATH1	Lstr026777.1	Chr2 24427309-24428678	134	15600.07	9.51	MATH-only
LsMATH2	Lstr032436.1	Chr8 9181257-9215428	1115	128995.2	5.42	MATH-USP7
LsMATH3	Lstr033584.1	Chr8 10618312-10627939	413	46590.65	6.39	MATH-BTB
LsMATH4	Lstr013707.1	ChrX 2204417-2214099	384	43487.70	5.87	MATH-BTB
LsMATH5	Lstr018574.1	Chr0 Contig253 6892-26389	1011	110188.97	5.70	MATH-Zf-Box

Table S3. Primers used for experiment

Primers	Sequences (5’-3’)
*NlMATH1*_F	GTTGGAGGGAAGTGGAGTT
*NlMATH1*_R	CAGTAGTAGCGTGAGGTTGG
*NlMATH2*_F	CTGAACCCAGTCAACGAA
*NlMATH2*_R	CCCAGATGCGGAACATAC
*NlMATH3*_F	GGTGCTTACGTGTTAATCCA
*NlMATH3*_R	TGAACAAACCTGTAGGCTCT
*NlMATH4*_F	GACGGTGGTGTAAGTTCG
*NlMATH4*_R	GCATCTGATAGATCGGGTA
ds*NlMATH3*_F	GGATCCTAATACGACTCACTATAGGGGTGCTTACGTGTTAATCCA
ds*NlMATH3*_R	GGATCCTAATACGACTCACTATAGGTGAACAAACCTGTAGGCTCT
Q*NlVg*-F	GCATCAATGAACCCAGCTAACTC
Q*NlVg*-R	TGGACGGCTCTTTGCATACTCC
Q*NlVgR*-F	AGGCAGCCACACAGATAACCGC
Q*NlVRg*-R	AGCCGCTCGCTCCAGAACATT
LOC111064163-F	ATCTTGGAGGTGAAGACTTTG
LOC111064163-R	CTGGGTTGATTGATAGGTTG
LOC120349581-F	TGACGGATGATAAAGAGGAC
LOC120349581-R	TGACTTGGTAACAGGCACTA
LOC111062677-F	CGACAACAAGAAGGACGAAAG
LOC111062677-R	CCTACTACAGGAGGAGGCAGATT
LOC111064220-F	GAACCATCCGACAGTCTACG
LOC111064220-R	TGCTTGCCTTCAATCACAAT
LOC111064087-F	AGCAACGACCAACAGACACG
LOC111064087-R	GACCTTGGGCGAGGAACTGG
LOC111059901-F	TGAAAGCCCTACTCCACCAT
LOC111059901-R	AGAACCGGACTCACATGCTC

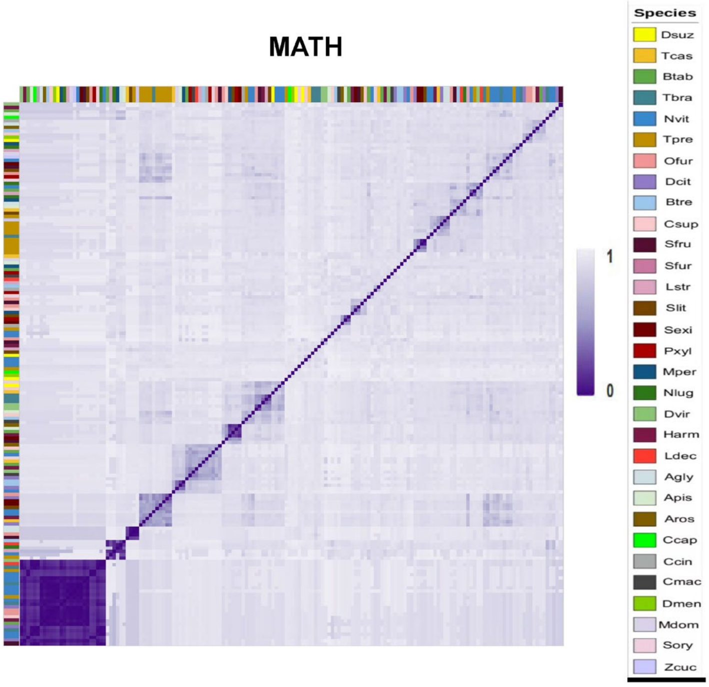

Figure S1. Structural similarity matrix to reflect similarities 121 identified MATH memebrs across 31 insect species.

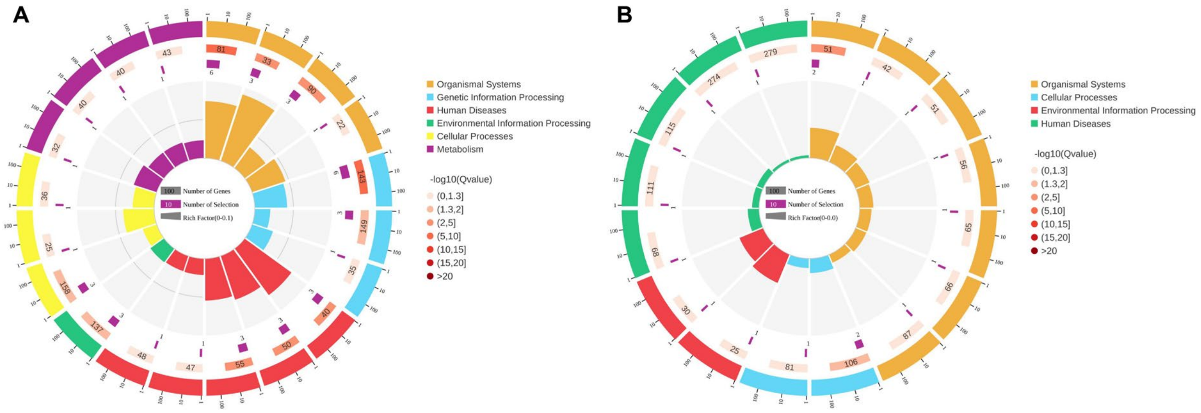

Figure S2. Transcriptomic analysis of *N. lugens* females after *NlMATH3* silencing. (A-B) Kyoto Encyclopedia of Genes and Genomes (KEGG) pathway enrichment analysis of differentially expressed genes (DEGs) that were upregulated (A) and downregulated (B) by RNA interference for 3 days.
